# A multicenter retrospective study of nivolumab monotherapy in previously treated metastatic renal cell carcinoma patients: interim analysis of Japanese real-world data

**DOI:** 10.1007/s10147-020-01692-z

**Published:** 2020-06-09

**Authors:** Nobuyuki Hinata, Junji Yonese, Satoru Masui, Yasutomo Nakai, Suguru Shirotake, Katsunori Tatsugami, Teruo Inamoto, Masahiro Nozawa, Kosuke Ueda, Toru Etsunaga, Takahiro Osawa, Motohide Uemura, Go Kimura, Kazuyuki Numakura, Kazutoshi Yamana, Hideaki Miyake, Satoshi Fukasawa, Kenya Ochi, Hirokazu Kaneko, Hirotsugu Uemura

**Affiliations:** 1 Division of Urology, Department of Surgery Related, Kobe University Graduate School of Medicine, Kobe, Japan; 2 Department of Urology, Cancer Institute Hospital of JFCR, Tokyo, Japan; 3 Division of Reparative and Regenerative Medicine, Nephro-Urologic Surgery and Andrology, Institute of Medical Life Science, Mie University Graduate School of Medicine, Mie, Japan; 4 Department of Urology, Osaka International Cancer Institute, Osaka, Japan; 5 Department of Uro-Oncology, Saitama Medical University International Medical Center, Saitama, Japan; 6 Department of Urology, Kyushu University Graduate School of Medical Sciences, Fukuoka, Japan; 7 Department of Urology, Osaka Medical College, Osaka, Japan; 8 Department of Urology, Kindai University, Faculty of Medicine, 377-2, OhnoHigashi, Osakasayama-shi, Osaka, 589-8511 Japan; 9 Department of Urology, Kurume University School of Medicine, Fukuoka, Japan; 10 Department of Urology, Isesaki Municipal Hospital, Gunma, Japan; 11 Department of Urology, Hokkaido University Graduate School of Medicine, Hokkaido, Japan; 12 Department of Urology, Osaka University Graduate School of Medicine, Osaka, Japan; 13 Department of Urology, Nippon Medical School Hospital, Tokyo, Japan; 14 Department of Urology, Akita University Graduate School of Medicine, Akita, Japan; 15 Department of Urology, Niigata University Graduate School of Medical and Dental Sciences, Niigata, Japan; 16 Department of Urology, Hamamatsu University School of Medicine, Shizuoka, Japan; 17 Prostate Center and Division of Urology, Chiba Cancer Center, Chiba, Japan; 18 Ono Pharmaceutical Co., Ltd, Osaka, Japan; 19 Bristol-Myers Squibb K.K, Tokyo, Japan

**Keywords:** Efficacy, Japan, Metastatic renal cell carcinoma, Nivolumab, Real-world, Safety

## Abstract

**Background:**

In a phase III clinical trial, CheckMate 025, treatment of metastatic renal cell carcinoma (mRCC) with nivolumab demonstrated superior efficacy over everolimus. However, as the clinical trial excluded patients with specific complications and poor performance status (PS), the effectiveness and safety of nivolumab in clinical practice, in which patients with various clinical complications are treated, is unclear. This study explored real-world nivolumab treatment in Japanese mRCC patients.

**Methods:**

This is an interim analysis of a multicenter, non-interventional, medical record review study (minimum follow-up: 9 months). All eligible Japanese mRCC patients who first received nivolumab between February and October 2017 were included; data cut-off was April 2019. We analyzed nivolumab treatment patterns, efficacy (including overall survival, progression-free survival, objective response rate, and duration of response) and safety (including immune-related adverse events).

**Results:**

Of 208 evaluable patients, 31.7% received nivolumab as fourth- or later line of treatment. At data cut-off, 26.9% of patients were continuing nivolumab treatment. The major reason for discontinuation was disease progression (*n* = 100, 65.8%). Median overall survival was not reached; the 12-month survival rate was 75.6%. Median progression-free survival was 7.1 months, the objective response rate was 22.6%, and median duration of response was 13.3 months. Patients who were excluded or limited in number in CheckMate 025, such as those with non-clear cell RCC or poor PS, also received benefits from nivolumab treatment. Immune-related adverse events occurred in 27.4% of patients (grade ≥ 3, 10.1%).

**Conclusion:**

Nivolumab was effective and well-tolerated in real-world Japanese mRCC patients.

**Trial registration:**

UMIN000033312

**Electronic supplementary material:**

The online version of this article (10.1007/s10147-020-01692-z) contains supplementary material, which is available to authorized users.

## Introduction

Renal cell carcinoma (RCC) occurs mostly in people between the ages of 50 and 70 years, and in about twice as many men as women [1, 2]. A majority of patients are diagnosed when the tumor is still relatively localized and amenable to surgical removal [3]. Importantly, the incidence of RCC increases with age, making it a major healthcare issue in countries with an aging society, like Japan [2]. An analysis of treatment patterns (2012–2015) among 277 Japanese patients indicated that most patients with metastatic (m)RCC received tyrosine kinase inhibitors (TKIs; 72.2%) and mammalian target of rapamycin inhibitors (mTORis; 14.3%) as first-line therapy. TKI–TKI treatment represents the most commonly used sequence (58.8%), and TKI-mTORi is the second most common (14.1%). Shorter duration of first-line treatment with TKIs results in poorer prognosis [4]. Thus, there is a clear need for improved therapeutic options.

Recently, the focus of mRCC treatment research has moved to immuno-oncology, and evaluations of immune-checkpoint inhibitors have shifted the treatment paradigm of mRCC [5, 6]. Nivolumab is a fully human monoclonal IgG4 antibody specific for the programmed death-1 cell surface receptor [7]. In a randomized phase III clinical trial (CheckMate 025), nivolumab was shown to be superior to everolimus in patients with previously treated advanced RCC [8]. Thus, nivolumab is the first drug that has been shown to prolong overall survival (OS) in treated mRCC patients. Based on these data, in 2016, nivolumab as a single agent was approved in Japan for the treatment of patients with unresectable RCC or mRCC who have received prior therapy [9]. Nivolumab is currently recommended by the Japanese Urological Association (JUA) renal cancer guideline for second-line therapy after progression on a TKI and for third-line therapy after failure of two TKIs [10].

However, the limited current knowledge about nivolumab use in Japanese patients with mRCC highlights two major concerns. One is that CheckMate 025 excluded patients with non-clear cell (ncc)RCC and enrolled a limited number of patients with Eastern Cooperative Oncology Group performance status (ECOG PS) of ≥ 2, those with brain metastasis or decreased renal function or those who were elderly [8]. The other is the small number of Japanese patients (*n* = 37) in the nivolumab group in CheckMate 025 [11, 12].

In addition to insufficient clinical trial data, there is little real-world evidence in Japanese patients. While there are several reports from other countries [13–16] and some analyses of patient groups excluded from CheckMate 025 [17], no similar multicenter or large-scale analyses have been reported in Japan. This clinical study was planned to analyze the treatment patterns of nivolumab for mRCC patients in clinical practice, and the efficacy and safety of nivolumab for these patients, by retrospective analyses of information from medical records. The study is ongoing, and this article focuses on interim analysis data.

## Patients and methods

### Patients

All patients with mRCC (diagnosed according to JUA guidelines [10]) who first received nivolumab during the period from 1 Feb 2017 to 31 Oct 2017, regardless of the treatment line, were included in this study. This interim analysis focused on patients with follow-up data for at least 6 months after treatment administration. Exclusion criteria were age < 20 years, previous participation in any clinical trial of any anticancer agents before or after nivolumab treatment, or participation in a nivolumab regulatory post-marketing surveillance study (JapicCTI-184069).

### Study design

This is an ongoing multicenter, retrospective, non-interventional, medical record review study, conducted at 17 hospitals in Japan. Data cut-off for this interim analysis was 26 April 2019. Data collection from patient medical records was planned at two-time points: between August 2018 and April 2019 (follow-up of ≥ 9 months after the first nivolumab treatment), and between November and December 2020 (follow-up of ≥ 36 months after the first nivolumab treatment). Baseline data were collected between the time of the initial diagnosis of mRCC and immediately before the start of systemic chemotherapy.

### Ethics

This study is being conducted in compliance with all appropriate national and international ethical guidelines and with the Act of Protection of Personal Information. The Ethics Committee at each site reviewed and approved the study protocol and all related documentation. All patients were given the opportunity to reject study participation (opt-out); written informed consent was required by the Ethics Committees at some study sites.

### Endpoints

In this study, we evaluated the treatment pattern of nivolumab in real-world clinical settings (including treatment history before and after nivolumab, treatment period, and treatment line), the 1-year OS rate, and nivolumab efficacy [progression-free survival (PFS), best overall response (BOR), objective response rate (ORR), duration of response (DOR), and disease control rate (DCR)] and adverse events (AEs) including immune-related (ir) AEs. Additional evaluations included subgroup analyses based on patient characteristics, treatment history, and occurrence of irAEs (event type, grade, and treatment).

### Statistical methods

The efficacy population included all eligible patients who met the study criteria, and the safety population included all enrolled patients who received treatment with nivolumab. OS was defined as the period from the date of first nivolumab administration to the date of death (or to the data cut-off date for this analysis, in case of ongoing survival). PFS was defined as the period from the date of first nivolumab administration to the date of either initial disease progression or death, or to the data cut-off date. DOR was defined as the period from the date of best response [complete or partial response (CR/PR)] during nivolumab administration to the earliest date of confirmed progressive disease (PD) or death, start date of the next treatment, or to the data cut-off date. ORR was defined as the proportion of patients with CR and PR as the best response; DCR was the proportion of patients with CR, PR or stable disease (SD) as the best treatment response. OS, BOR, DOR, and PD were based on investigator assessments per Response Evaluation Criteria in Solid Tumors (RECIST) version 1.1. AEs were coded using the Medical Dictionary for Regulatory Activities version 21.1. Severity was classified based on the Common Terminology Criteria for Adverse Events version 4.0.

For OS and PFS, graphical outputs were created based on the Kaplan–Meier methodology. The survival rate for each month was calculated; the median of each endpoint was calculated with their 95% confidence intervals (CI). For other parameters, quantitative variables were summarized using descriptive statistics, and categorical variables were summarized using number and percentage. A swimmer plot provided a visual representation of nivolumab treatment duration, BOR, PD, death, and reason for discontinuation. Logistic regression analysis was conducted to estimate the odds ratio of response and its 95% CI (calculated using the Chi-square test). Variables included age, tissue type, ECOG PS, International Metastatic RCC Database Consortium (IMDC) risk, Karnofsky performance status (KPS) < 80%, hemoglobin below the lower limit of normal, corrected serum calcium ≥ 10 mg/dL, the period from RCC diagnosis to treatment start date < 1 year, neutrophils at or above the upper limit of normal (≥ ULN), platelets ≥ ULN, irAEs, TKI resistance, neutrophil–lymphocyte ratio, lactate dehydrogenase (LDH), serum albumin, C-reactive protein, and estimated glomerular filtration rate. SAS version 9.4 (SAS Institute, Cary, NC, USA) was used for statistical calculations.

## RESULTS

### Patients

In total, 208 patients who met enrollment criteria were analyzed for efficacy and safety. Table 1 shows baseline demographics and clinical characteristics of patients at the start of nivolumab treatment. Approximately three-quarters of patients were male (76.0%), and the mean age was 66.5 years. The majority had an ECOG PS of 0 or 1 (*n* = 120, 57.7%) and a diagnosis of ccRCC (*n* = 160, 76.9%). Of the patients with nccRCC, the subtypes included papillary (*n* = 10), chromophobe (*n* = 2), spindle cell (*n* = 5), and other (*n* = 31). The most common metastasis site was lung (*n* = 155, 74.5%), and 172 patients (82.7%) had a history of nephrectomy. KPS was < 80% in 12.5% of patients, and 23.1% had a poor IMDC risk.

**Table 1:** Baseline demographic and clinical characteristics

Variable	Patients
Total	208 (100.0)
Sex	
Male	158 (76.0)
Female	50 (24.0)
Age at the start of nivolumab administration (years)	
Mean (standard deviation)	66.5 (10.1)
< 65	73 (35.1)
65–74	92 (44.2)
≥ 75	43 (20.7)
ECOG PS	
0	70 (33.7)
1	50 (24.0)
2	16 (7.7)
3 or 4	10 (4.8)
Unknown	62 (29.8)
Tissue type	
Clear	160 (76.9)
Non-clear	48 (23.1)
Papillary	10 (20.8)
Chromophobe	2 (4.2)
Spindle cell	5 (10.4)
Other	31 (64.6)
Lung metastasis	
Yes	155 (74.5)
Liver metastasis	
Yes	34 (16.3)
Bone metastasis	
Yes	73 (35.1)
Brain metastasis	
Yes	13 (6.3)
Lymph node metastasis	
Yes	77 (37.0)
Other metastasis	
Yes	90 (43.3)
IMDC risk	
Favorable (0 risk)	21 (10.1)
Intermediate (1 risk)	66 (31.7)
Intermediate (2 risks)	72 (34.6)
Poor (≥ 3 risks)	48 (23.1)
KPS < 80% at the start of nivolumab administration	
Yes	26 (12.5)
Unknown	61 (29.3)
Hemoglobin < LLN	
Yes	147 (70.7)
Unknown	2 (1.0)
Corrected serum calcium ≥ 10 mg/dL	
Yes	26 (12.5)
Unknown	11 (5.3)
Period from RCC diagnosis to treatment start date < 1 year	
Yes	113 (54.3)
Unknown	1 (0.5)
Neutrophils ≥ ULN	
Yes	46 (22.1)
Unknown	6 (2.9)
Platelets ≥ ULN	
Yes	26 (12.5)
Unknown	2 (1.0)
Nephrectomy^a^	
Yes	172 (82.7)
Treatment duration of first-line TKI followed by second-line nivolumab	
< 6 months	39 (18.8)
≥ 6 months	36 (17.3)
NLR	
< 5	129 (62.0)
≥ 5	33 (15.9)
LDH (IU/L)	
< 207.8	101 (48.6)
≥ 207.8	67 (32.2)
Albumin(g/dL)	
< 3.34	70 (33.7)
≥ 3.34	90 (43.3)
CRP (mg/dL)	
< 0.8	85 (40.9)
≥ 0.8	81 (38.9)
eGFR (mL/min/1.73 m^2^)^b^	
< 60	135 (64.9)
≥ 60	35 (16.8)

Data are shown as *n* (%) unless otherwise specified

*CRP* C-reactive protein, *ECOG PS* Eastern Cooperative Oncology Group performance status, *eGFR* estimated glomerular filtration rate, *IMDC* International Metastatic RCC Database Consortium, *KPS* Karnofsky performance status, *LDH* lactate dehydrogenase, *LLN* lower limit of normal, *NLR* neutrophil–lymphocyte ratio, *RCC* renal cell carcinoma, *TKI* tyrosine kinase inhibitor, *ULN* upper limit of normal

^a^Radical nephrectomy and/or partial nephrectomy

^b^Percentage calculated from evaluable patients

### Treatment patterns

Table 2 displays nivolumab treatment patterns. The median number of nivolumab administrations at the time of data cut-off was 12 (range 1–47), and the median duration of treatment was 6.3 months (range 0.0–24.7). Nivolumab was administered as first-line treatment in two patients (1.0%), as second-line in 76 patients (36.5%), as third-line in 64 patients (30.8%), and as fourth- or later line in 66 patients (31.7%). Both before and after nivolumab treatment, TKIs were the most commonly used therapeutic agents (90.4% and 31.3%, respectively). At the time of data cut-off, 56 patients (26.9%) were continuing nivolumab treatment. The major reason for discontinuation was disease progression (*n* = 100, 65.8%).

**Table 2:** Real-world nivolumab treatment patterns

Factor	
Patients, *N*	208
Number of doses, median (range)	12 (1–47)
Duration of treatment (months), median (range)	6.3 (0.0–24.7)
Treatment line^a^, *n* (%)	
1st	2 (1.0)
2nd	76 (36.5)
3rd	64 (30.8)
≥ 4th	66 (31.7)
Ongoing treatment, *n* (%)	56 (26.9)
Discontinuation of treatment, *n* (%)	152 (73.1)
Reason for discontinuation of treatment^b^, *n* (%)	
Progression of mRCC	100 (65.8)
AE and/or ADR	43 (28.3)
Discontinuation after confirming efficacy	1 (0.7)
Patient request	10 (6.6)
Death	9 (5.9)
Status immediately before nivolumab therapy^c^	
Classification, therapeutic drugs, *n* (%)	
VEGFR-TKI	188 (90.4)
mTORi	13 (6.3)
Cytokine	2 (1.0)
Others	3 (1.4)
Status immediately after nivolumab therapy	
Classification, therapeutic drugs, *n* (%)	
VEGFR-TKI	65 (31.3)
mTORi	8 (3.8)
Cytokine	0 (0.0)
Others	0 (0.0)
No treatment	135 (64.9)
Ongoing nivolumab	56 (41.5)
No treatment after nivolumab therapy	79 (58.5)

*ADR* adverse drug reaction, *AE* adverse event, *mTORi* mammalian target of rapamycin inhibitor, *mRCC* metastatic renal cell carcinoma, *VEGFR-TKI* vascular endothelial growth factor receptor-tyrosine kinase inhibitor

^a^All patients received TKI as perioperative treatment

^b^Multiple answers were allowed

^c^Included patients who received nivolumab as second- or later line of therapy

### Efficacy outcomes

**Fig. 1:**
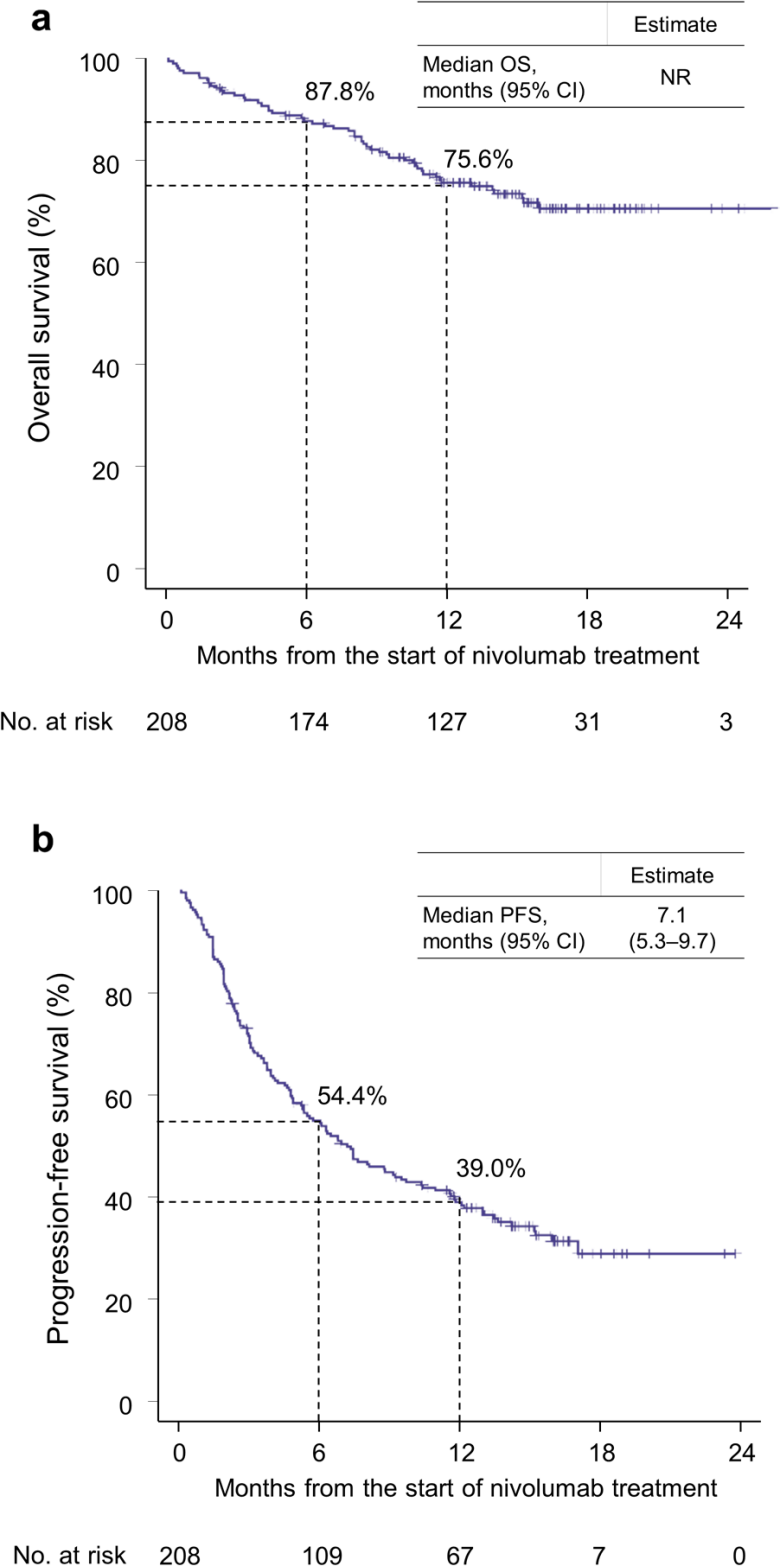
Kaplan–Meier estimate of **a** overall survival and **b** progression-free survival. *CI* confidence interval, *NR* not reached, *OS* overall survival, *PFS* progression-free survival

In this interim analysis, the median OS was not reached. The 1-year survival rate (*n* = 127) was 75.6% (95% CI 69.0–81.1) (Fig. 1a), and median PFS was 7.1 months (95% CI 5.3–9.7) (Fig. 1b).

**Fig. 2:**
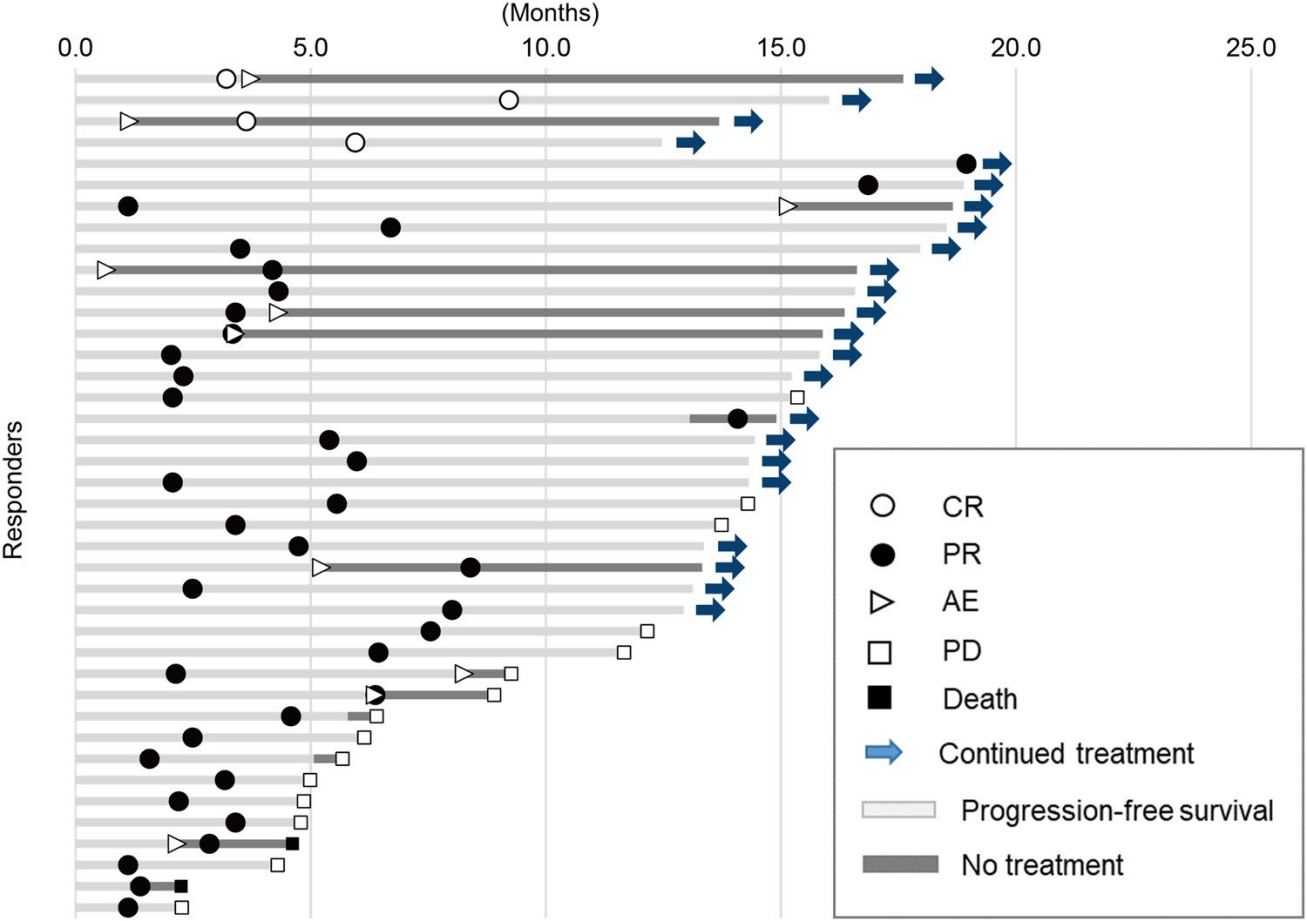
Treatment duration in patients who responded to nivolumab. *AE* adverse event, *CR* complete response, *PD* progressive disease, *PR* partial response.

The ORR was 22.6%, with four patients (2.3%) achieving CR and 36 patients (20.3%) achieving PR; the DCR was 61.0%, and median DOR was 13.3 months (range 5.2–NE) (Table 3). Among responders, 17 patients (42.5%) discontinued nivolumab, mostly due to progression; however, 23 patients (57.5%) showed persistent response for more than 1 year with continued treatment (Fig. 2).

**Table 3:** Best overall response

Variable	*N* = 208
Assessment of BOR	
* n* (%)	177 (85.1)
BOR^a^	
CR	4 (2.3)
PR	36 (20.3)
SD	68 (38.4)
PD	69 (39.0)
ORR^a^	
* n* (%)	40 (22.6)
95% CI	(16.7–29.5)
DCR^a^	
* n* (%)	108 (61.0)
95% CI	(53.4–68.2)

*BOR* best overall response, *CI* confidence interval, *CR* complete response, *DCR* disease control rate, *ORR* objective response rate, *PD* progressive disease, *PR* partial response, *RECIST* response evaluation criteria in solid tumors, *SD* stable disease

^a^Calculated from patients who had an assessment of BOR made by investigators, per RECIST version 1.1

### Additional efficacy evaluations

**Fig. 3:**
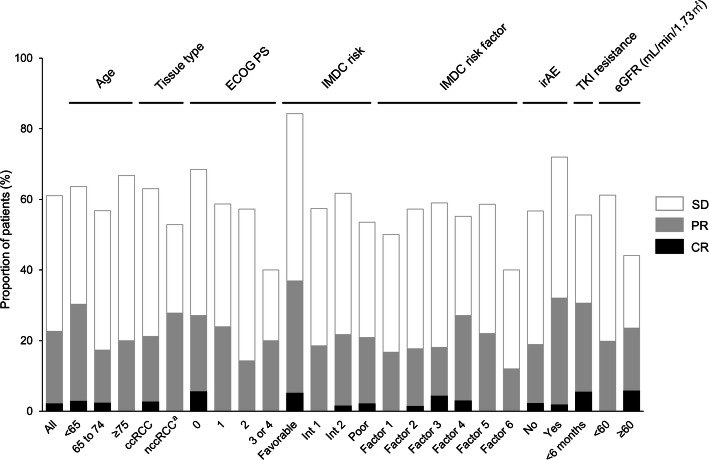
BOR by subgroup. ^a^Overall response rate by nccRCC subtype: papillary 12.5% (1/8), chromophobe 0% (0/2), spindle cell 40% (2/5), and other 33.3% (7/21). *BOR* best overall response, *ccRCC* clear cell renal cell carcinoma, *CR* complete response, *ECOG PS* Eastern Cooperative Oncology Group performance status, *eGFR* estimated glomerular filtration rate, *IMDC* International Metastatic RCC Database Consortium, *Factor *1 Karnofsky performance status < 80%, *Factor* 2 hemoglobin < LLN, *Factor* 3 corrected serum calcium ≥ 10 mg/dL, *Factor* 4 period from RCC diagnosis to treatment start date < 1 year, *Factor* 5 neutrophils ≥ ULN, *Factor* 6 platelets ≥ ULN, *Int* 1 intermediate (1 risk factor), *Int* 2 intermediate (2 risk factors), *irAE* immune-related adverse event, *LLN* lower limit of normal, *nccRCC* non-clear cell renal cell carcinoma *PR* partial response, *SD* stable disease, *TKI* tyrosine kinase inhibitor, *ULN* upper limit of normal

In subgroup analyses according to patient background factors, PFS was significantly improved in patients with lower ECOG PS (*P* = 0.0082) but was unaffected by age, tissue type, IMDC risk, and TKI resistance (Online Resource 1a-1e). In univariate analysis, ECOG PS, KPS, rates of irAEs, and levels of platelets, LDH, and serum albumin were all significantly associated with PFS (Table 4, Online Resource 2a). In multivariate analysis, ECOG PS, and levels of platelets and LDH remained associated with PFS (Table 4, Online Resource 2b). BOR by subgroup is shown in Fig. 3.

**Table 4:** Effectiveness according to patient background factors: univariate and multivariate analyses for progression-free survival

Factor	Variable	Reference	Univariate		Multivariate	
			HR (95% CI)	*P* value	HR (95% CI)	*P* value
Age (years)	65–74	< 65	0.98 (0.68–1.42)	0.9255	–	–
	≥ 75	< 65	0.61 (0.37–1.01)	0.0543	–	–
Tissue type	Non-clear	Clear	1.24 (0.84–1.84)	0.2819	–	–
ECOG PS	2, 3, 4	0, 1	2.15 (1.35–3.44)	0.0013	2.28 (1.26–4.12)	0.0064
IMDC risk	Int1	Favorable	1.32 (0.69–2.52)	0.3976	–	–
	Int2	Favorable	1.36 (0.72–2.57)	0.3468	-	–
	Poor	Favorable	2.06 (1.07–3.98)	0.0304	–	-
KPS < 80%	Yes	No	2.18 (1.37–3.48)	0.0011	-	-
Hemoglobin < LLN	Yes	No	1.32 (0.90–1.93)	0.1583	–	–
Corrected serum calcium ≥ 10 mg/dL	Yes	No	0.81 (0.47–1.41)	0.4627	–	–
Period from RCC diagnosis to treatment start date < 1 year	Yes	No	1.16 (0.82–1.63)	0.4078	–	–
Neutrophils ≥ ULN	Yes	No	1.36 (0.91–2.03)	0.1278	–	–
Platelets ≥ ULN	Yes	No	2.65 (1.70–4.15)	< 0.0001	2.01 (1.11–3.63)	0.0207
irAE	Yes	No	0.63 (0.42–0.95)	0.0276	0.81 (0.47–1.41)	0.4616
TKI resistance	≥ 6 months	< 6 months	0.87 (0.49–1.54)	0.6394	–	–
NLR	≥ 5	< 5	1.55 (1.00–2.41)	0.0510	–	–
LDH (IU/L)	≥ 207.8	< 207.8	1.89 (1.30–2.75)	0.0008	1.72 (1.08–2.72)	0.0211
Albumin (g/dL)	≥ 3.34	< 3.34	0.58 (0.40–0.85)	0.0048	0.78 (0.47–1.28)	0.3208
CRP (mg/dL)	≥ 0.8	< 0.8	1.20 (0.82–1.75)	0.3396	–	–
eGFR (mL/min/1.73m^2^)	≥ 60	< 60	1.54 (0.99–2.38)	0.0547	–	–

*CI* confidence interval, *CRP* C-reactive protein, *ECOG PS* Eastern Cooperative Oncology Group performance status, *eGFR* estimated glomerular filtration rate, *HR* hazard ratio, *IMDC* International Metastatic RCC Database Consortium, *Int*1 intermediate (1 risk), *Int*2 intermediate (2 risks), *irAE* immune-related adverse event, *KPS* Karnofsky performance status, *LDH* lactate dehydrogenase, *LLN* lower limit of normal, *NLR* neutrophil–lymphocyte ratio, *RCC* renal cell carcinoma, *TKI* tyrosine kinase inhibitor, *ULN* upper limit of normal

### Safety outcomes

AEs are summarized in Table 5. Fifty-seven patients (27.4%) reported at least one irAE, of which the most frequent were endocrine disorders (7.2%) and pulmonary toxicity (5.3%). Just 21 patients (10.1%) reported severe irAEs with a grade of ≥ 3, of which seven (3.4%) were pulmonary toxicity.

**Table 5:** Summary of immune-related adverse events

	Number of patients, (%) (*N* = 208)	
	Any grade	Grade ≥ 3
Any AE	159 (76.4)	90 (43.3)
Any irAE	57 (27.4)	21 (10.1)
Endocrine disorder	15 (7.2)	3 (1.4)
Skin toxicity	10 (4.8)	2 (1.0)
Pulmonary toxicity	11 (5.3)	7 (3.4)
Hepatotoxicity	6 (2.9)	2 (1.0)
Gastrointestinal toxicity	10 (4.8)	1 (0.5)
Nervous system disorder	2 (1.0)	1 (0.5)
Nephrotoxicity	5 (2.4)	2 (1.0)
Muscle disorder	3 (1.4)	0 (0.0)
Eye disorder	3 (1.4)	0 (0.0)
Blood toxicity	2 (1.0)	2 (1.0)
Metabolism and nutrition disorders	3 (1.4)	2 (1.0)
Others	9 (4.3)	0 (0.0)

*AE* adverse event, *irAE* immune-related adverse event

**Fig. 4:**
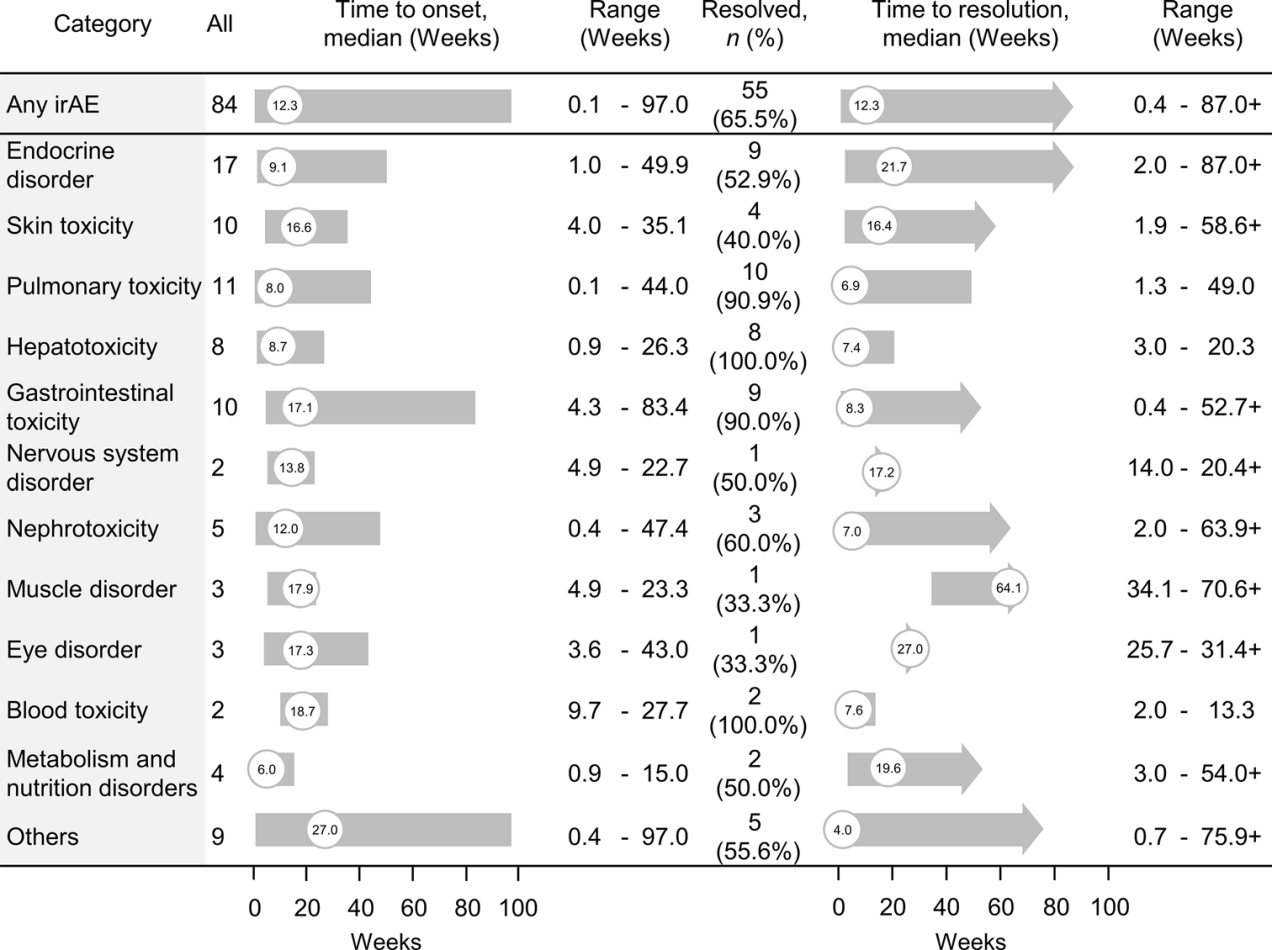
Time to onset and time to resolution of irAEs. Median is shown in a circle. Symbol + indicates a censored value *irAE* immune-related adverse event

The median time to onset of irAEs was 12.3 weeks overall, the median time to resolution was also 12.3 weeks, and 65.5% of irAEs were resolved (Fig. 4). Pulmonary toxicity, nephrotoxicity, and hepatotoxicity resolved in 6.9, 7.0, and 7.4 weeks, respectively. The steroid usage rate in patients with irAEs was 50.9%.

Nivolumab treatment modifications for patients who experienced irAEs are described in Online Resource 3. A total of 84 irAE events were reported, of which 74 irAE events were shown during nivolumab administration. Of 43 irAE events that resulted in nivolumab treatment suspension, 19 events (44.2%) were followed by nivolumab administration being restarted. Of those 19 irAE events, one (5.3%) subsequently relapsed (hepatotoxicity). Of 31 irAE events during which nivolumab treatment continued, three (9.7%) resulted in treatment discontinuation/suspension after some time.

## Discussion

The results of our analysis indicate that nivolumab efficacy was consistent between the clinical trial setting and real-world clinical practice. Compared with CheckMate 025 [8], nivolumab-treated patients in our study were older (mean age 62 vs. 66.5 years, respectively) and had worse KPS (5.9% vs. 12.5%, respectively, had a score of < 80%). Frequencies of lung, bone, and brain metastases were also higher in our study. Furthermore, patients in our study were heavily treated, with almost one-third (31.7%) receiving nivolumab as fourth- or later-line of treatment for mRCC, and 23.1% having a poor IMDC risk.

Despite these patient characteristics, nivolumab demonstrated good clinical outcomes in our study. In this interim analysis, the 12-month survival rate was high, suggesting long term efficacy. However, the median OS could not be determined in the current study due to the short observation period, and thus, it is difficult to directly compare with CheckMate 025. Conversely, the median PFS in this analysis was higher than that in the CheckMate 025 global [8] and Japanese [11, 12] populations (7.1 vs. 4.6 vs. 5.6 months, respectively). Moreover, 57.5% of responders had CR/PR, which persisted for more than 1 year, providing further evidence for the long-term efficacy of nivolumab. The current study also indicated that, in the Japanese real-world treatment situation, patients are likely to receive longer durations of nivolumab treatment compared with a clinical trial (the median duration of treatment in our study was 6.3 months vs. 5.5 months in CheckMate 025 [8]). In addition, there were no new safety signals in our analysis compared with previous reports of nivolumab treatment [18, 19].

CheckMate 025 excluded patients with nccRCC. In comparison, 23.1% of patients in the current analysis had nccRCC. Nivolumab treatment in these patients appeared to be effective, and consistent efficacy was observed in the subgroup analyses regardless of tissue type; the overall response rate by subtype was papillary 12.5% (1/8), chromophobe 0% (0/2), spindle cell 40% (2/5), and other 33.3% (7/21), supporting the potential to treat a broader population of mRCC patients in this study. Our data obtained from nccRCC patients are consistent with the recent report of the CheckMate 374 study, in which clinically meaningful antitumor activity was reported in patients with advanced or metastatic nccRCC [17].

It has been reported that patients treated with TKI as first-line therapy for less than 6 months (defined as ‘TKI-resistant’) have a poor prognosis and that subsequent therapies are less effective [5]. The current analysis could suggest that both TKI-resistant (first-line TKI duration < 6 months) and TKI-non-resistant patients (first-line TKI duration ≥ 6 months) may obtain benefit from nivolumab treatment because PFS (4.8 months vs. 7.0 months) and ORR (16.7% vs. 30.6%) with nivolumab were comparable (Online Resource 1e).

IMDC is commonly used as a prognostic factor in renal cell carcinoma [20]. In our subgroup analyses, all IMDC risk classes had similar ORR, but platelet level was an independent risk factor for PFS. Similarly, ECOG PS has also been used as a prognostic factor in other carcinomas [21]. In this analysis, ECOG PS was considered as an independent risk factor for PFS, but the lack of significant differences indicated that it was not a risk factor for ORR. Multivariate analyses showed the benefit of nivolumab was obtained in patients with good ECOG PS, platelet < ULN and lower LDH. However, it is unclear whether patients with poor ECOG PS, platelet ≥ ULN and higher LDH treated with nivolumab get more benefit than those treated with TKI or mTORi. In other carcinomas, the onset of irAEs has been reported to be associated with improvements in PFS and OS [22]. Consistent with this, in our study, a high ORR and prolonged PFS were observed in irAE-expressing patients, although the presence of irAEs was not an independent risk factor. However, in this analysis, the relationship between the onset time of irAE and efficacy for nivolumab is unclear.

Several irAEs, including pulmonary toxicity, gastrointestinal toxicity, nephrotoxicity, blood toxicity, and metabolism and nutrition disorders generally resulted in discontinuation of nivolumab treatment, whereas more than half of patients with an endocrine disorder and skin toxicity continued nivolumab treatment with appropriate manipulations (Online Resource 3). Thus, it is important for oncologists to manage irAEs properly.

## Limitations

This study has several limitations, including the retrospective, observational design, and the small number of study sites, which may be insufficient to accurately reflect the entire Japanese mRCC population. The short observation period was another limitation of this study. As in other retrospective observational studies, only the data entered into the medical records are available for analysis, and no additional information can be obtained. Therefore, some records might be improperly collected, and the required information might be missing. These limitations may lead to underestimation and/or overestimation during the resultant analyses.

## Conclusions

Nivolumab was effective and well-tolerated in the Japanese real-world setting, with outcomes consistent with the results of the CheckMate 025 clinical trial. No new safety signals were observed. Real-world nivolumab efficacy was found to be similar across all patient subpopulations, even those with poor prognosis who were not included in the clinical trial population. Long-term prognostic data will continue to be collected in this ongoing study and will be reported in a future publication.

## Electronic supplementary material

Below is the link to the electronic supplementary material.

Supplementary file Online Resource 1 (PDF 222 kb)

Supplementary file Online Resource 2 (PDF 177 kb)

Supplementary file Online Resource 3 (PDF 71 kb)
