# Supplementary material for: A multicenter retrospective study of nivolumab monotherapy in previously treated metastatic renal cell carcinoma patients: interim analysis of Japanese real-world data
Source: Int J Clin Oncol. 2020 Jun 9;25(8):1533–42. doi: 10.1007/s10147-020-01692-z (PMC7392942; doi:10.1007/s10147-020-01692-z)
Supplement: Supplementary file 1 — Supplementary file Online Resource 1 (PDF 222 kb) [file 10147_2020_1692_MOESM1_ESM.pdf]

# A multicenter retrospective study of nivolumab monotherapy in previously treated metastatic renal cell carcinoma patients: Interim analysis of Japanese real-world data

*International Journal of Clinical Oncology*

Nobuyuki Hinata, Junji Yonese, Satoru Masui, Yasutomo Nakai, Suguru Shirotake, Katsunori Tatsugami, Teruo Inamoto, Masahiro Nozawa, Kosuke Ueda, Toru Etsunaga, Takahiro Osawa, Motohide Uemura, Go Kimura, Kazuyuki Numakura, Kazutoshi Yamana, Hideaki Miyake, Satoshi Fukasawa, Kenya Ochi, Hirokazu Kaneko, and Hirotsugu Uemura

**Corresponding author**
Name: Hirotsugu Uemura
Address: Department of Urology, Kindai University Faculty of Medicine, 377-2, OhnoHigashi, Osakasayama-shi, Osaka 589-8511, Japan.
Tel: +81-72-366-0221
Fax: +81-72-365-6273
E-mail: huemura@med.kindai.ac.jp

**Online resource 1** Kaplan–Meier estimates of PFS by subgroup. (a) age, (b) tissue type, (c) ECOG PS, (d) IMDC risk, and (e) TKI resistance.

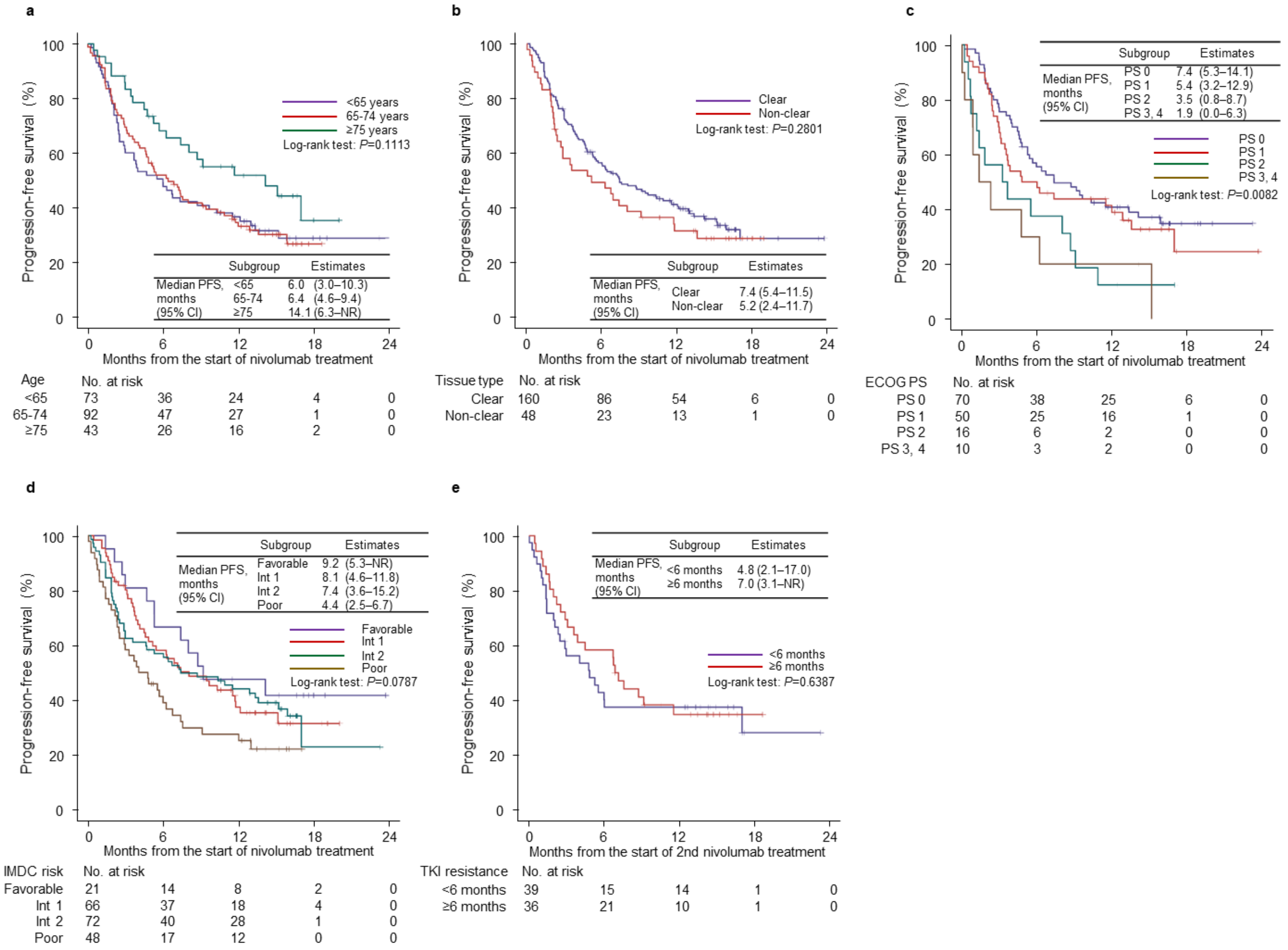


CI, confidence interval; ECOG PS, Eastern Cooperative Oncology Group performance status; IMDC, International Metastatic RCC Database Consortium; Int 1, intermediate (1 risk); Int 2, intermediate (2 risks); NR, not reached; PFS, progression-free survival; TKI, tyrosine kinase inhibitor.
