# Supplementary material for: A multicenter retrospective study of nivolumab monotherapy in previously treated metastatic renal cell carcinoma patients: interim analysis of Japanese real-world data
Source: Int J Clin Oncol. 2020 Jun 9;25(8):1533–42. doi: 10.1007/s10147-020-01692-z (PMC7392942; doi:10.1007/s10147-020-01692-z)
Supplement: Supplementary file 2 — Supplementary file Online Resource 2 (PDF 177 kb) [file 10147_2020_1692_MOESM2_ESM.pdf]

A multicenter retrospective study of nivolumab monotherapy in previously treated metastatic renal cell carcinoma patients: Interim analysis of Japanese real-world data

International Journal of Clinical Oncology

Nobuyuki Hinata, Junji Yonese, Satoru Masui, Yasutomo Nakai, Suguru Shirotake, Katsunori Tatsugami, Teruo Inamoto, Masahiro Nozawa, Kosuke Ueda, Toru Etsunaga, Takahiro Osawa, Motohide Uemura, Go Kimura, Kazuyuki Numakura, Kazutoshi Yamana, Hideaki Miyake, Satoshi Fukasawa, Kenya Ochi, Hirokazu Kaneko, and Hirotsugu Uemura

Corresponding author
Name: Hirotsugu Uemura
Address: Department of Urology, Kindai University Faculty of Medicine, 377-2, OhnoHigashi, Osakasayama-shi, Osaka 589-8511, Japan.
Tel: +81-72-366-0221
Fax: +81-72-365-6273
E-mail: huemura@med.kindai.ac.jp

Online Resource 2. Forest plots showing (a) univariate and (b) multivariate analyses of PFS by subgroup.

a

Factor	Variable	Reference	Univariate	
			HR (95% CI)	*P*-value
Age (years)	65-74	<65	0.98 (0.68-1.42)	0.9255
	≥ 75	<65	0.61 (0.37-1.01)	0.0543
Tissue type	Non-clear	Clear	1.24 (0.84-1.84)	0.2819
ECOG PS	2, 3, 4	0, 1	2.15 (1.35-3.44)	0.0013
IMDC risk	Int1	Favorable	1.32 (0.69-2.52)	0.3976
	Int2	Favorable	1.36 (0.72-2.57)	0.3468
	Poor	Favorable	2.06 (1.07-3.98)	0.0304
Factor 1	Yes	No	2.18 (1.37-3.48)	0.0011
Factor 2	Yes	No	1.32 (0.90-1.93)	0.1583
Factor 3	Yes	No	0.81 (0.47-1.41)	0.4627
Factor 4	Yes	No	1.16 (0.82-1.63)	0.4078
Factor 5	Yes	No	1.36 (0.91-2.03)	0.1278
Factor 6	Yes	No	2.65 (1.70-4.15)	<0.0001
irAE	Yes	No	0.63 (0.42-0.95)	0.0276
TKI resistance	≥6 months	<6 months	0.87 (0.49-1.54)	0.6394
NLR	≥5	<5	1.55 (1.00-2.41)	0.0510
LDH (IU/L)	≥207.8	<207.8	1.89 (1.30-2.75)	0.0008
Albumin (g/dL)	≥3.34	<3.34	0.58 (0.40-0.85)	0.0048
CRP (mg/dL)	≥0.8	<0.8	1.20 (0.82-1.75)	0.3396
eGFR (mL/min/1.73m^2)	≥ 60	<60	1.54 (0.99-2.38)	0.0547

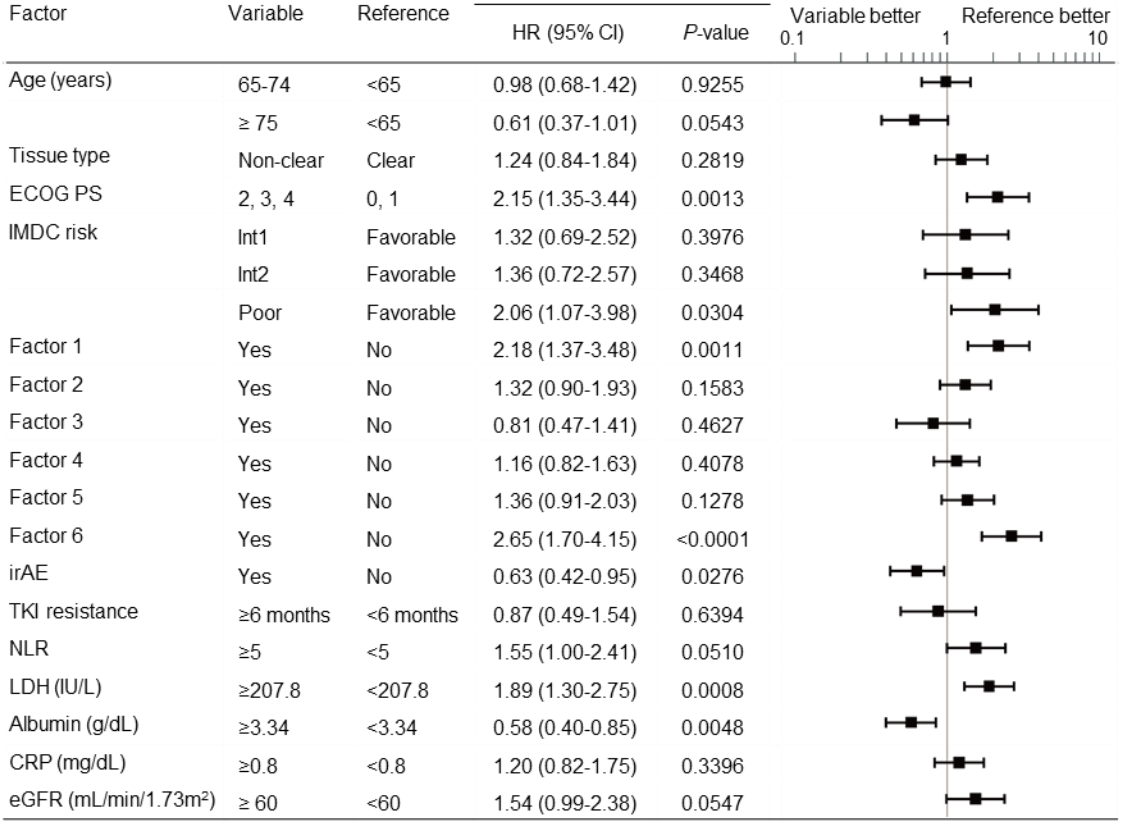

b

Factor	Variable	Reference	Multivariate	
			HR (95% CI)	*P*-value
ECOG PS	2, 3, 4	0, 1	2.28 (1.26-4.12)	0.0064
Factor 6	Yes	No	2.01 (1.11-3.63)	0.0207
irAE	Yes	No	0.81 (0.47-1.41)	0.4616
LDH (IU/L)	≥207.8	<207.8	1.72 (1.08-2.72)	0.0211
Albumin (g/dL)	≥3.34	<3.34	0.78 (0.47-1.28)	0.3208

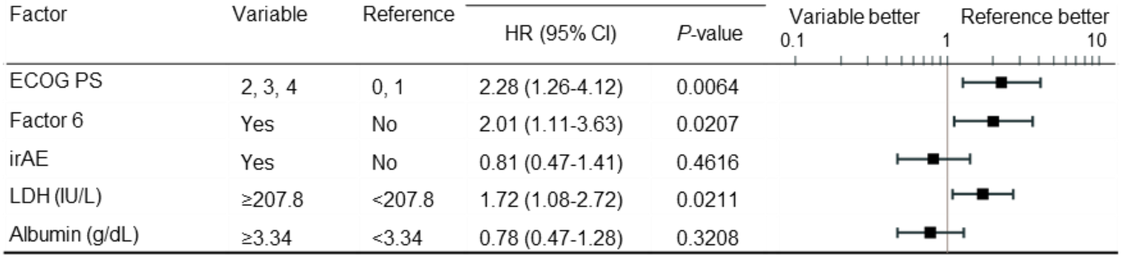

CI, confidence interval; CRP, C-reactive protein; ECOG PS, Eastern Cooperative Oncology Group performance status; eGFR, estimated glomerular filtration rate; Factor 1, Karnofsky performance status <80%; Factor 2,

hemoglobin <LLN; Factor 3, corrected serum calcium ≥10mg/dL; Factor 4, period from RCC diagnosis to treatment start date <1 year; Factor 5, neutrophils ≥ULN; Factor 6, platelets ≥ULN; HR, hazard ratio; IMDC, International Metastatic RCC Database Consortium; Int1, intermediate (1 risk); Int2, intermediate (2 risks); irAE, immune-related adverse event; LDH, lactate dehydrogenase; LLN, lower limit of normal; NLR, neutrophil-lymphocyte ratio; RCC, renal cell carcinoma; TKI, tyrosine kinase inhibitor; ULN, upper limit of normal.
