# Supplementary material for: A multicenter retrospective study of nivolumab monotherapy in previously treated metastatic renal cell carcinoma patients: interim analysis of Japanese real-world data
Source: Int J Clin Oncol. 2020 Jun 9;25(8):1533–42. doi: 10.1007/s10147-020-01692-z (PMC7392942; doi:10.1007/s10147-020-01692-z)
Supplement: Supplementary file 3 — Supplementary file Online Resource 3 (PDF 71 kb) [file 10147_2020_1692_MOESM3_ESM.pdf]

A multicenter retrospective study of nivolumab monotherapy in previously treated metastatic renal cell carcinoma patients: Interim analysis of Japanese real-world data

International Journal of Clinical Oncology

Nobuyuki Hinata, Junji Yonese, Satoru Masui, Yasutomo Nakai, Suguru Shirotake, Katsunori Tatsugami, Teruo Inamoto, Masahiro Nozawa, Kosuke Ueda, Toru Etsunaga, Takahiro Osawa, Motohide Uemura, Go Kimura, Kazuyuki Numakura, Kazutoshi Yamana, Hideaki Miyake, Satoshi Fukasawa, Kenya Ochi, Hirokazu Kaneko, and Hirotsugu Uemura

Corresponding author
Name: Hirotsugu Uemura
Address: Department of Urology, Kindai University Faculty of Medicine, 377-2, OhnoHigashi, Osakasayama-shi, Osaka 589-8511, Japan.
Tel: +81-72-366-0221
Fax: +81-72-365-6273
E-mail: huemura@med.kindai.ac.jp

Online Resource 3. Nivolumab treatment modifications for patients who experienced irAEs. (a) by class of irAE, (b) overall.

a

		Number of patients with irAE	Number of irAE events	Number of irAEs during nivolumab administration	Number of discontinuations and drug holidays	Number of administration restarts	irAE re-onset	Continued administration of nivolumab	Discontinuation and drug holiday due to irAE	irAE after discontinuation of nivolumab
Number of Patients		208 (100.0%)	-	-	-	-	-	-	-	-
irAE	Yes	57 (27.4%)	84	74 (88.1%)	43 (58.1%)	19 (44.2%)	1 (5.3%)	31 (41.9%)	3 (9.7%)	10 (11.9%)
	Endocrine disorder	15 (7.2%)	17	17 (100.0%)	5 (29.4%)	2 (40.0%)	0 (0.0%)	12 (70.6%)	0 (0.0%)	0 (0.0%)
	Skin toxicity	10 (4.8%)	10	10 (100.0%)	3 (30.0%)	3 (100.0%)	0 (0.0%)	7 (70.0%)	0 (0.0%)	0 (0.0%)
	Pulmonary toxicity	11 (5.3%)	11	9 (81.8%)	8 (88.9%)	2 (25.0%)	0 (0.0%)	1 (11.1%)	0 (0.0%)	2 (18.2%)
	Hepatotoxicity	6 (2.9%)	8	5 (62.5%)	3 (60.0%)	1 (33.3%)	1 (100.0%)	2 (40.0%)	0 (0.0%)	3 (37.5%)
	Gastrointestinal toxicity	10 (4.8%)	10	8 (80.0%)	7 (87.5%)	3 (42.9%)	0 (0.0%)	1 (12.5%)	0 (0.0%)	2 (20.0%)
	Nervous system disorder	2 (1.0%)	2	2 (100.0%)	1 (50.0%)	0 (0.0%)	0 (0.0%)	1 (50.0%)	1 (100.0%)	0 (0.0%)
	Nephrotoxicity	5 (2.4%)	5	4 (80.0%)	4 (100.0%)	1 (25.0%)	0 (0.0%)	0 (0.0%)	0 (0.0%)	1 (20.0%)
	Muscle disorder	3 (1.4%)	3	3 (100.0%)	2 (66.7%)	1 (50.0%)	0 (0.0%)	1 (33.3%)	0 (0.0%)	0 (0.0%)
	Eye disorder	3 (1.4%)	3	3 (100.0%)	0 (0.0%)	0 (0.0%)	0 (0.0%)	3 (100.0%)	2 (66.7%)	0 (0.0%)
	Blood toxicity	2 (1.0%)	2	1 (50.0%)	1 (100.0%)	1 (100.0%)	0 (0.0%)	0 (0.0%)	0 (0.0%)	1 (50.0%)
	Metabolism and nutrition disorder	3 (1.4%)	4	4 (100.0%)	3 (75.0%)	2 (66.7%)	0 (0.0%)	1 (25.0%)	0 (0.0%)	0 (0.0%)
	Other	9 (4.3%)	9	8 (88.9%)	6 (75.0%)	3 (50.0%)	0 (0.0%)	2 (25.0%)	0 (0.0%)	1 (11.1%)

b

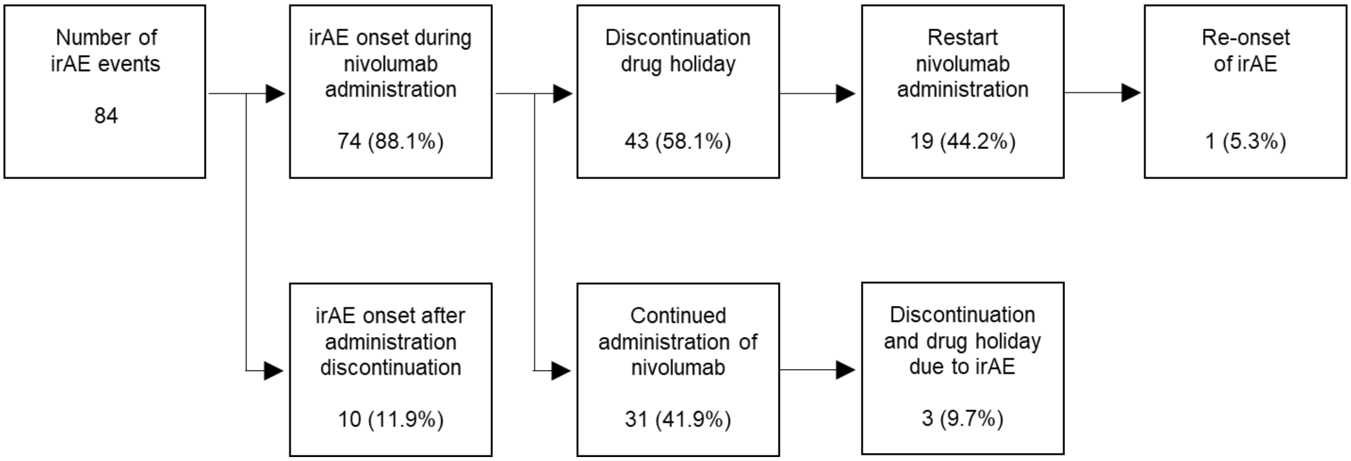

Data are shown as *n* (%).

irAE, immune-related adverse event.
